# Structural and functional cardiac parameters across occupations: a cross-sectional study in differing work environments

**DOI:** 10.1038/s41598-024-62190-0

**Published:** 2024-05-27

**Authors:** Roman Leischik, Peter Foshag, Chayakrit Krittanawong, Ulrich Jehn, Richard Vollenberg, Markus Strauss

**Affiliations:** 1 Department of Cardiology, Faculty of Health, School of Medicine, University Witten/Herdecke, 58455 Witten, Germany; 2 Cardiology Division, NYU Langone Health and NYU School of Medicine, New York, NY USA; 3 Department of Medicine D, Division of General Internal Medicine, Nephrology and Rheumatology, University Hospital of Muenster, 48149 Muenster, Germany; 4 Department of Medicine B, Gastroenterology and Hepatology, University Hospital Münster, 48149 Muenster, Germany; 5 Department of Cardiology I, Coronary and Peripheral Vascular Disease, Heart Failure Medicine, University Hospital Muenster, Cardiol, Albert-Schweitzer-Campus 1, 48149 Muenster, Germany

**Keywords:** Cardiac function, Cardiovascular risk, Echocardiography, Firefighters, Police officers, Office workers, Cardiovascular diseases, Cardiology, Health occupations, Risk factors

## Abstract

Previous investigations have highlighted notable variations in cardiovascular risk indicators associated with various professional categories. However, only a few studies have examined structural and functional cardiac parameters using echocardiography within distinct occupational groups. Hence, this study endeavored to assess cardiac structural and functional parameters in three additional occupations: firefighters (FFs), police officers (POs), and office workers (OWs). This prospective study encompassed 197 male participants (97 FFs, 54 POs, and 46 OWs) from Germany. All participants underwent 2D and Doppler echocardiography in resting conditions; standard parasternal and apical axis views were employed to evaluate structural (diastolic and systolic) and functional (systolic and diastolic function, and strain) cardiac parameters. All three occupational groups exhibited a tendency towards septal hypertrophy. Notably, OWs exhibited the largest diastolic interventricular septum diameter (IVSd), at 1.33 ± 0.25 cm. IVSd significantly varied between POs and OWs (*p* = 0.000) and between POs and FFs (*p* = 0.025). Additionally, during diastole a substantially larger left ventricular posterior wall diameter (LVPWd) was observed in OWs compared to FFs (*p* = 0.001) and POs (*p* = 0.013). The left ventricular diastolic cavity diameter (LVIDd) and the left ventricular systolic cavity diameter (LVIDs) were significantly higher in POs than they were in FFs (LVIDd: *p* = 0.001; LVIDs: *p* = 0.009), and the LVIDd was notably higher in FFs (*p* = 0.015) and POs compared to OWs (p = 0.000). FFs exhibited significantly better diastolic function, indicated by higher diastolic peak velocity ratios (MV E/A ratio) and E/E’ ratios, compared to POs (E/A ratio: *p* = 0.025; E/E’ ratio: *p* = 0.014). No significant difference in diastolic performance was found between OWs and FFs. Significantly higher E’(lateral) values were noted in POs compared to FFs (*p* = 0.003) and OWs (*p* = 0.004). Ejection fraction did not significantly differ among FFs, POs, and OWs (*p* > 0.6). The left ventricular mass (LV Mass) was notably higher in POs than it was in FFs (*p* = 0.039) and OWs (*p* = 0.033). Strain parameter differences were notably improved in two- (*p* = 0.006) and four-chamber (*p* = 0.018) views for FFs compared to POs. Concentric remodeling was the predominant change observed in all three occupational groups. Significant differences in the presence of various forms of hypertrophy were observed in FFs, POs, and OWs (exact Fisher test p-values: FFs vs. OWs = 0.021, POs vs. OWs = 0.002). OWs demonstrated notably higher rates of concentric remodeling than FFs did (71.77% vs. 47.9%). This study underscores disparities in both functional and structural parameters in diverse occupational groups. Larger prospective studies are warranted to investigate and delineate differences in structural and functional cardiac parameters across occupational groups, and to discern their associated effects and risks on the cardiovascular health of these distinct professional cohorts.

Health risk profile variations are well-documented across distinct occupational groups^1–3^. Väisänen et al.^4^ illustrated significant disparities in health risk markers across occupational divisions; in particular, they reported a higher aggregation of health risks in blue-collar workers compared to their white-collar counterparts in an occupational health service screening involving over 72,000 participants. Within the international security realm, two crucial occupational groups, firefighters (FFs) and police officers (POs), both operate in shift patterns and face elevated health risks^5^. Conversely, office workers (OWs) typically endure sedentary working conditions that constitute up to 71% of their workday^6^. Such work-related sedentary activities are linked to increased cardiovascular risks^7^. The professional sector predominantly engaged in office work is one of the largest global occupational segments, with over 9 million individuals employed in office work in Germany alone^8^.

It has been established that office workers exhibit distinct health behaviors, often associated with a poorer health prognosis, compared to the general population^9^. Specifically, cross-national studies reveal that office workers tend to lead unhealthy lifestyles and are physically inactive^10^.

Cardiovascular diseases are the primary causes of on-duty fatalities among firefighters^11^. Moreover, the amalgamation of cardiovascular risk factors with extreme physical workloads precipitates cardiovascular complications such as sudden cardiac death, myocardial infarction, and stroke^12^. Research indicates that high physical and psychological demands are placed on FFs and POs^11,13–16^. Their shift-based working conditions also pose health hazards^17^. Previous studies of German FFs and POs indicated that a significant proportion of participants faced unexpectedly high health risks^18–20^. French firefighter studies also identified considerable cardiovascular risks, and identified obesity and arterial hypertension as significant risk factors^21^.

A limited number of studies have investigated echocardiography parameters across various occupations. Moreover, minimal information exists regarding the disparity in structural and functional cardiac parameters among three occupational groups: FFs, POs, and OWs. For example athletes are one of the best examined group for structural changes of the left ventricular function during exercise^22^. Echocardiography is a fundamental diagnostic tool used to detect early cardiac dysfunction, and offers vital support and management for cardiovascular patients in the workplace^23^. Hence, this study aimed to examine these distinct occupational groups’ cardiac structural and functional parameters to ascertain any trends indicative of cardiac dysfunction.

## Methods

### Study population

We included 197 male participants (97 FFs, 54 POs, and 46 OWs) in the present study. Invitations to participate in this study were made via the internet, social media, and local corporate distribution after participants responded to an official request. FFs worked primarily in a German fire departments’ fire service. POs were all employed in service and patrol duty in a German federal police force. Participating OWs were administration employees in public administration or financial management; inclusion criteria included a predominantly sedentary occupation in an administrative office.

Participants underwent physical examination and 2D and Doppler echocardiography. In cases of evident clinical illness, medical reports were sent to participants’ family doctors.

### Ethical statement

The Ethics Committee of the University of Witten/Herdecke (application no. 121/2013) approved this study. Participation was voluntary, and each study participant provided written informed consent. All analysis was performed in accordance with relevant regulations of the committee and the Declaration of Helsinki. All participants signed written, informed consent.

### Echocardiography

Echocardiographic examinations were performed by medical physicians with specific echocardiography evaluation training using a commercial ultrasound machine (Vivid E 9 Dimension®, General Electrics (GE) Systems) equipped with an S3 probe (2–4 MHz). Two-dimensional assessments of left ventricular cavity diameter, wall thickness, and mass were performed according to the criteria of the American Society of Echocardiography and the European Association of Cardiovascular Imaging^24^. Two-dimensional greyscale M-mode and B-mode images were recorded in standard parasternal short- and long-axis views as well as in apical four-, three-, and two-chamber views. Images were obtained at the level of the left ventricle and the aortic valve to assess aortic root diameter (Ao); left atrial diameter (LA); left atrial volume (LA volume); fractional shortening (%FS); left ventricular diastolic (LVIDd) and left ventricular systolic (LVIDs) cavity diameters; left diastolic and systolic ventricular posterior wall (LVPWd and LVPWs) diameters; diastolic interventricular septum diameter (IVSd); systolic interventricular septum diameter (IVSs); left ventricular mass (LV mass); left ventricular end-diastolic volume (LVEDV); left ventricular end-systolic (LVESV) volume; ejections fraction (EF); and stroke volume (SV).

Left ventricular ejection fraction (LVEF) was measured using Simpson's rule and biplane images from apical four- and two-chamber views. LV mass was determined using Devereux's formula. To detect hypertrophy types, the criteria of the American Society of Echocardiography and the European Association of Cardiovascular Imaging were used^24^. We used pulsed-wave (PW) Doppler to record left ventricular velocities from the apical four-chamber view. We positioned the PW-Doppler sample at the mitral leaflets' tip, with the ultrasound parallel to the flow stream. At this position, we measured early (MV E Max) and late (MV A-Max) diastolic peak velocity and their ratios (MV E/A ratio). We measured E' as an early diastolic peak (E' (lateral)) using Doppler tissue imaging (DTI) signals to record PW Doppler in the apical four-chamber view, positioned in the myocardium within the basal lateral wall, within 1 cm of the mitral annulus. Next, we determined the E/E' ratio.

Strain echocardiography is a well introduced and reproducible method for assessment of cardiac function^25,26^.Strain and strain rate data were recorded in apical four-, two-, and three-chamber views using the Vivid E 9 Dimension von GE Ultrasound. Afterwards, technicians used GE Medical Systems to perform offline strain analysis on a separate workstation using EchoPAC Dimension software (product version 112, 1.5, 298). Apical four-, two-, and three-chamber views were recorded using a high frame rate via entry through the left ventricle in the echocardiographic plane. We intentionally chose cardiac cycles of the same length during the respiratory phase of expiration. The operator performing the analysis was not the echocardiography investigator and was blinded to participants’ data. Semiautomatic border detection was used to identify the interface between the left ventricular walls and cavity for the analysis. When bad borders were recognized, technicians performed manual corrections to ensure correct tracing of the endocardial and epicardial border and corrected the wrong segment of the left ventricular. In this study, we recorded apical longitudinal four-, two-, and three-chamber views (to assess four-chamber strain, two-chamber strain, and three-chamber strain) via automated function imaging (AFI). Global strain (average strain) was calculated by averaging all regional values of peak systolic deformation determined in each segment of the three apical views before aortic valve closure in a seventeen-segment left ventricle model. Furthermore, we used 2D Speckle tracking to measure the apical four-chamber view's longitudinal strain (LV) and peak systolic strain rate (LV longitudinal strain rate). Participants’ strain data were excluded from the analysis when three or more myocardial segments could not be evaluated.

### Blood pressure, nicotine consumption, work experience and sports activity

The measurement of blood pressure was performed in a supine position with calibrated standard blood pressure cuffs*.* We used a questionnaire to collect *work experience, medical history information, sports activity (h/week), corporate sports (h/week),* cigarette consumption (number of cigarettes per day) and to calculate the metabolic equivalents (METS) based on Ainsworth et al.^27^. Precisely and alcohol consumption (alcohol consumption in several days per week) were surveyed. Dynamic sports activities such as jogging, cycling, swimming, soccer, *strength training* were assessed for one week. One MET corresponds to 1 kcal·kg^−1^·h^−1^.

### Statistical analysis

Data description was separate for each of the three groups. Anthropometric and echocardiographic parameters were defined using means (MW) and standard deviations (SD). Differences between groups were estimated using a linear regression adjusted for age because most analyzed parameters were directly age-related; 95% confidence intervals (CIs) were also reported. All statistical tests were two-sided with a 0.05 significance level. Due to this study's exploratory nature, *p*-values were not adjusted for multiple testing. We used Stata/IC 13.1 for Windows for statistical analysis.

## Results

### Anthropometric and body characteristics, medical history

Anthropometric and basic characteristics of FFs, POs, and OWs are presented in Table 1. FFs were significantly younger than POs and OWs (OWs vs. FFs, *p* = 0.003; POs vs. FFs, *p* = 0.001). Therefore, further comparisons were adjusted for age between occupational groups. POs had the highest weight (93.6 ± 13.2 kg). FFs and OWs weighed significantly less than POs did (FFs, 85.9 ± 11.5 kg; OWs, 87.1 ± 13.4 kg). FFs had the lowest body fat percentage, which was significant compared to POs (*p* = 0.010). POs and OWs were obese, with a mean BMI > 25 mg/m^2^; BMI differences were significant between POs and OWs (*p* = 0.036) and POs and FFs (*p* = 0.005). Systolic (RRsys) and diastolic (RRdia) blood pressure, as well as nicotine onsumption, did not differ significantly between the groups. In accordance with the statistical calculations, there was no significant difference regarding hypertension between the POs and FFs groups, but a tendency towards higher blood pressure is evident in the POs (6.1% FFs vs. 21.7% OWs). One could already pose the question whether the little stronger hypertrophy in the POs is explainable by this tendency. Perhaps higher numbers of participants from the civil service sector would be needed to answer this question definitively.

**Table 1:** Anthropometric and basic characteristics of the study population (firefighters, police officers, and office workers).

Variables	Firefighters (FFs) (n = 97)	Police officers (POs) (n = 55)	Office workers (OWs) (n = 46)	Linear regression Estimated difference [95%-CI], *p*-value (adjusted for age)		
	Mean ± SD	Mean ± SD	Mean ± SD	OWs vs. FFs	POs vs. FFs	POs vs. OWs
Age	40.5 ± 9.0	45.3 ± 7.8	45.8 ± 10.0	5.30 (1.86–8.74) *p* = 0.003	4.82 (2.06–7.58) *p* = 0.001	−0.48 (−4.09–3.13) *p* = 0.792
Weight (kg)	85.9 ± 11.5	93.6 ± 13.2	87.1 ± 13.4	0.13 (−4.38–4.64) *p* = 0.955	6.59 (2.34–10.84) *p* = 0.003	6.67 (1.44–11.90) *p* = 0.013
Height (cm)	182.2 ± 6.3	182.8 ± 6.8	181.5 ± 5.4	−0.10 (−2.12–1.93) *p* = 0.924	1.12 (−1.02–3.26) *p* = 0.301	1.28 (−1.14–3.70) *p* = 0.296
Body mass index (kg/m^2^)	25.9 ± 3.2	28.0 ± 3.2	26.4 ± 4.1	0.08 (−1.22–1.38) *p* = 0.904	1.61 (0.51–2.71) *p* = 0.005	1.56 (0.11–3.02) *p* = 0.036
Body surface area	2.08 ± 0.16	2.18 ± 0.18	2.09 ± 0.17	0.00 (−0.06–0.06) *p* = 0.999	0.09 (0.03–0.14) *p* = 0.004	0.09 (0.02–0.16) *p* = 0.012
Body fat %	17.7 ± 6.2	21.4 ± 5.6	20.8 ± 6.5	2.05 (−0.23–4.33) *p* = 0.078	2.44 (0.61–4.28) *p* = 0.010	0.74 (−1.65–3.12) *p* = 0.541
RRsys (mmHg)	126.4 ± 9.8	127.7 ± 12.3	129.1 ± 11.7	2.19 (−1.81–6.19) *p* = 0.281	0.73 (−3.42–4.89) *p* = 0.728	−1.40 (−6.14–3.34) *p* = 0.558
RRdia (mmHg)	84.1 ± 7.4	85.7 ± 10.2	86.6 ± 8.7	1.55 (−1.34–4.44) *p* = 0.292	1.02 (−2.16–4.21) *p* = 0.526	−0.93 (−4.68–2.81) *p* = 0.621
Nicotine consumption (cig/day)	2.57 ± 6.26	2.52 ± 5.98	1.72 ± 5.53	−1.27 (−3.31–0.76) *p* = 0.219	−0.20 (−2.40–2.00) *p* = 0.857	0.82 (−1.45–3.09) *p* = 0.474

Regarding previous cardiometabolic history, 6.1% (n = 6) of the FFs, 14.8% (n = 8) of the POs and 21.7% (n = 10) of the OWs had a diagnosis of arterial hypertension. In one participant from the group of office workers, diabetes mellitus was present. In the other two professional groups, there was no diagnosis of diabetes mellitus. None of participants of all groups had a diagnosis of heart failure and coronary heart disease.

Table 2 shows the work experience and sports activity of the study population, measured in metabolic equivalents (METS). One MET corresponds to an energy consumption of 1 kcal per kgKG/h. Work experience in years was highest in the group of police officers. They had significantly more years of professional experience than FFs (*p* = 0.000) and OWs (*p* = 0.001). FFs did significantly more sport per week than OWs (*p* = 0.003). Firefighters achieved the highest number of hours of corporate sports per week. Regarding the proportion of hours of corporate sports per week, all groups differed significantly from one another. On average, FFs achieved the highest metabolic equivalents for all the sports listed and for the METS total. Significant differences were found in the METS for strength training between FFs and POs (*p* = 0.013) and between OWs and FFs (*p* = 0.025). These differences were also significant between the groups for cycling (OWs vs.FFs: *p* = 0.003 ; POs vs. FFs: *p* = 0.001).

**Table 2:** Work experience and sports activity of the study population (firefighters, police officers, and office workers).

Variables	Firefighters (FFs) (n = 97)	Police officers (POs) (n = 55)	Office workers (OWs) (n = 46)	Linear regression Estimated difference [95%-CI], p-value (adjusted for age)		
	Mean ± SD	Mean ± SD	Mean ± SD	OWs vs. FFs	POs vs. FFs	POs vs. OWs
Work experience (years)	16.3 ± 9.1	25.2 ± 8.4	21.1 ± 10.8	0.34 (−2.07–2.76) *p* = 0.779	4.51 (3.06–5.96) *p* = 0.000	4.48 (1.86–7.10) *p* = 0.001
Sport activity (h/week)	6.0 ± 3.6	4.83 ± 4.29	4.03 ± 3.02	−1.79 (−2.96–0.63) *p* = 0.003	−0.74 (−2.18–0.70) *p* = 0.312	0.74 (−0.68–2.17) *p* = 0.303
Corporate sports (h/week)	1.50 ± 1.52	0.58 ± 0.93	0.15 ± 0.63	−1.22 (−1.59–0.86) *p* = 0.000	−0.76 (−1.16–0.37) *p* = 0.000	0.42 (0.11–0.73) *p* = 0.009
Strength training (METS)	763 ± 833	594 ± 925	363.1 ± 652.1	−302.31 (−566.40–38.21) *p* = 0.025	−343.48 (−612.84− −74.12) *p* = 0.013	230.6 (−82–543) *p* = 0.146
Swimming (METS)	151.5 ± 300.4	77.9 ± 211.4	106.8 ± 280.8	−31.52 (−123.22–60.19) p = 0.499	−40.15 (−133.07− 52.77) p = 0.395	−28.9 (−129–71) p = 0.566
Soccer (METS)	261.3 ± 636.9	231.4 ± 621.5	117.3 ± 328.7	−101.10 (−265.62–63.42) *p* = 0.227	−117.17 (−288.75− 54.42) *p* = 0.180	114.1 (−78–306) *p* = 0.242
Jogging (METS)	977 ± 1161	900 ± 1072	703 ± 1280	−172.01 (−630.57–286.54) *p* = 0.460	−226.43 (−692.58− 239.72) *p* = 0.339	196.7 (−275–668) *p* = 0.410
Cycling (METS)	1436 ± 1944	802 ± 1754	713 ± 1139	−822.43 (−1361.18–283.68) *p* = 0.003	−920.54 (−1473.07—368.01) *p* = 0.001	89.0 (−487–665) *p* = 0.760

### Structural echocardiographic parameters

All three groups presented a septum hypertrophy tendency. OWs had the largest IVSd, with a 1.33 ± 0.25 cm septum thickness. The IVSd was significantly statistically different between POs and OWs (*p* = 0.000) and between POs and FFs (*p* = 0.025). The IVSs differed (not considerably) between the three occupational groups. Significantly larger LVPWds were detected in OWs compared to FFs (*p* = 0.001) and POs (*p* = 0.013). LVPWs did not significantly differ between groups. LVIDd and LVIDs diameters were markedly higher in POs than in FFs (LVIDd, *p* = 0.001; LVIDs, *p* = 0.009). The LVIDd was significantly higher in FFs (*p* = 0.015) and POs than it was in OWs (*p* = 0.000). Left atrial volume was significantly greater in POs than in OWs (*p* = 0.040). The LA diameters of the other groups as well as the LA volumes did not differ significantly between the groups. The reason why the POs exhibit a higher LA volume than the POS can only be speculated: perhaps it is a combination of individual factors, such as the tendency towards slightly more physical activity at the professional level (corporate sports).

Structural echocardiographic parameters are described in Table 3.

**Table 3:** Structural echocardiographic parameters of the study population (firefighters, police officers, and office workers).

Variables	Firefighters (FFs) (n = 97)	Police officers (POs) (n = 55)	Office workers (OWs) (n = 46)	Linear regression Estimated difference [95%-CI], p-value (adjusted for age)		
	Mean ± SD	Mean ± SD	Mean ± SD	OWs vs. FFs	POs vs. FFs	POs vs. OWs
LA (cm)	3.47 ± 0.39	3.53 ± 0.31	3.56 ± 0.44	0.04 (−0.11–0.19) *p* = 0.634	0.03 (−0.09–0.16) *p* = 0.624	−0.03 (−0.18–0.13) *p* = 0.725
LA volume (ml)	35 ± 10.0	38.6 ± 10.0	33.6 ± 12.9	−2.03 (−6.40–2.35) *p* = 0.361	3.25 (−0.37–6.86) *p* = 0.078	4.94 (0.24–9.64) *p* = 0.040
IVSd (cm)	1.21 ± 0.25	1.16 ± 0.18	1.33 ± 0.25	0.07 (−0.02–0.15) *p* = 0.120	−0.09 (−0.16–0.01) *p* = 0.025	−0.17 (−0.25–0.08) *p* = 0.000
IVSs (cm)	1.71 ± 0.30	1.73 ± 0.24	1.78 ± 0.28	0.02 (−0.08–0.11) *p* = 0.703	−0.02 (−0.10–0.07) *p* = 0.716	−0.04 (−0.14–0.05) *p* = 0.347
% IVS Thickness	42.7 ± 19.6	52.1 ± 20.4	36.1 ± 17.8	−5.76 (−12.43–0.91) *p* = 0.090	9.85 (3.13–16.57) *p* = 0.004	16.15 (8.46–23.85) *p* = 0.000
LVPWd (cm)	1.08 ± 0.19	1.10 ± 0.19	1.20 ± 0.17	0.12 (0.05–0.18) *p* = 0.001	0.04 (−0.03–0.10) *p* = 0.245	−0.09 (−0.16–0.02) *p* = 0.013
LVPWs (cm)	1.66 ± 0.24	1.67 ± 0.23	1.71 ± 0.21	0.06 (−0.02–0.14) *p* = 0.127	0.02 (−0.06–0.11) *p* = 0.593	−0.04 (−0.13–0.05) *p* = 0.349
% LVPW Thickness	56.1 ± 23.6	54.8 ± 29.0	45.8 ± 23.0	−8.68 (−17.53–0.17) *p* = 0.055	−1.61 (−10.99–7.77) *p* = 0.735	8.66 (−1.72–19.03) *p* = 0.101
LVIDd (cm)	4.78 ± 0.45	5.1 ± 0.6	4.49 ± 0.50	−0.22 (−0.40–0.04) *p* = 0.015	0.32 (0.13–0.51) *p* = 0.001	0.56 (0.35–0.78) *p* = 0.000
LVIDs (cm)	3.19 ± 0.40	3.39 ± 0.48	3.70 ± 4.43	0.36 (−0.58–1.30) *p* = 0.451	0.21 (0.05–0.36) *p* = 0.009	−0.28 (−1.52–0.95) *p* = 0.648

### Diastolic function

Each group’s diastolic parameters are displayed in Table 4. Firefighters (FFs) demonstrated a significantly better diastolic function, which was indicated by E/A and E/E′ ratios that were higher than those of police officers (POs) (E/A ratio, *p* = 0.025; E/E' ratio, *p* = 0.015). No significant difference in diastolic performance was observed between office workers (OWs) and FFs. A tendency towards a higher E/A ratio was observed in FFs compared to OWs (*p* = 0.051), although no significant difference in E/E′ ratio was evident (*p* = 0.200). Remarkably higher E'(lateral) values were noted in POs compared to FFs (*p* = 0.005) and OWs (*p* = 0.005). FFs’ maximum A wave was significantly higher than POs’ (*p* = 0.001) and OWs’ (*p* = 0.000) A waves were.

**Table 4:** Diastolic function parameters of the study population (firefighters, police officers, and office workers).

Variables	Firefighters (FFs) (n = 97)	Police officers (POs) (n = 55)	Office workers (OWs) (n = 46)	Linear regression Estimated difference [95%-CI], p-value (adjusted for age)		
	Mean ± SD	Mean ± SD	Mean ± SD	OWs vs. FFs	POs vs. FFs	POs vs. OWs
MV E Max (m/s)	0.67 ± 0.11	0.67 ± 0.13	0.68 ± 0.13	0.03 (−0.01–0.08) *p* = 0.098	0.02 (−0.02–0.06) *p* = 0.365	−0.01 (−0.06–0.03) *p* = 0.528
MV A Max (m/s)	0.48 ± 0.09	0.55 ± 0.11	0.56 ± 0.10	0.06 (0.03–0.10) *p* = 0.000	0.06 (0.02–0.09) *p* = 0.001	−0.01 (−0.05–0.03) *p* = 0.689
MV E/A Ratio	1.44 ± 0.28	1.26 ± 0.30	1.26 ± 0.31	−0.09 (−0.18–0.00) *p* = 0.051	−0.10 (−0.19–0.01) *p* = 0.025	−0.01 (−0.12–0.09) *p* = 0.834
E' (lateral) (m/s)	0.12 ± 0.03	0.13 ± 0.03	0.11 ± 0.03	−0.00 (−0.01–0.01) *p* = 0.701	0.02 (0.01–0.03) *p* = 0.003	0.02 (0.01–0.03) *p* = 0.004
E/E' Ratio (cm/s)	5.7 ± 1.5	5.2 ± 1.2	6.2 ± 1.4	0.35 (−0.19–0.90) *p* = 0.200	−0.61 (−1.09–0.12) *p* = 0.014	−0.93 (−1.46–0.40) *p* = 0.001

### Systolic function

The left ventricular end-diastolic volume (LVEDV) was significantly higher in FFs compared to POs (*p* = 0.018). However, there were no significant LVEDV differences between FFs and OWs, or between POs and OWs (*p* > 0.2). No significant differences in the left ventricular end-systolic volume (LVESV) were observed between participant groups (all *p*-values > 0.08). On average, all occupational groups exhibited a normal left ventricular ejection fraction (LVEF); there were no significant differences in ejection fraction between FFs, POs, and OWs (*p* > 0.5).

The left ventricular mass (LV Mass) was notably higher in POs compared to FFs (*p* = 0.015) and OWs (*p* = 0.015). Specifically, POs’ LV Mass measured 222 ± 20.5 g, and the relative left ventricular mass (g/m^2^) was 107 ± 25. Although there was a significantly higher absolute LV Mass in POs compared to FFs and OWs, the relative LV Mass (g/m^2^) did not exhibit substantial differences between groups (*p* > 0.1). Table 5 illustrates study participants’ systolic echocardiographic parameters.

**Table 5:** Systolic function parameters of the study population (firefighters, police officers, and office workers).

Variables	Firefighters (FFs) (n = 97)	Police officers (POs) (n = 55)	Office workers (OWs) (n = 46)	Linear regression Estimated difference [95%-CI], p-value (adjusted for age)		
	Mean ± SD	Mean ± SD	Mean ± SD	OWs vs. FFs	POs vs. FFs	POs vs. OWs
% FS	33.4 ± 4.9	33.1 ± 6.4	31.7 ± 3.8	−1.46 (−2.99–0.07) p = 0.061	−0.14 (−2.14–1.85) p = 0.886	1.31 (−0.77–3.40) p = 0.214
SV MOD A4C (ml)	67.9 ± 19.4	62.2 ± 16.9	63.7 ± 16.4	−4.59 (−11.11–1.92) p = 0.166	−5.24 (−11.57–1.09) p = 0.104	−1.52 (−8.25–5.21) p = 0.655
LVEF (%)	62.8 ± 6.6	62.5 ± 6.8	63.0 ± 5.7	0.31 (−1.96–2.58) p = 0.787	−0.04 (−2.40–2.31) p = 0.970	−0.49 (−2.98–2.00) p = 0.696
LVEDV MOD BP (ml)	106.2 ± 23.6	94.2 ± 19.7	98.2 ± 23.8	−5.10 (−13.58–3.37) p = 0.236	−8.29 (−15.12–1.47) p = 0.018	−4.06 (−12.92–4.80) p = 0.365
LVESV MOD BP (ml)	39.4 ± 10.9	35.4 ± 9.9	36.6 ± 11.3	−1.99 (−6.18–2.20) p = 0.350	−3.09 (−6.62–0.44) p = 0.085	−1.21 (−5.54–3.11) p = 0.579
LV Mass (ASE) (g)	211.5 ± 45.3	232.7 ± 60.5	212.1 ± 39.1	−1.44 (−15.51–12.63) p = 0.840	19.67 (1.04–38.29) p = 0.039	21.37 (1.72–41.02) p = 0.033
LV Mass (ASE) (g/m^2^)	101.4 ± 18.6	107.0 ± 25.3	101.8 ± 18.8	−0.08 (−6.53–6.36) p = 0.980	5.43 (−2.30–13.16) p = 0.167	5.52 (−3.16–14.20) p = 0.210

### Strain and strain rate parameters

Overall, FFs had the highest strain values in all chamber views (Table 6). FFs demonstrated significantly higher strain values in both two- (*p* = 0.006) and four-chamber views (*p* = 0.018) compared to POs. The two-chamber strain value was considerably higher in OWs and FFs than it was in POs (POs vs. FFs, *p* = 0.006; POs vs. OWs, *p* = 0.01). No significant differences in strain were observed between FFs and OWs in all chamber views. Additionally, there were no significant differences in three-chamber strain between groups (*p* > 0.3). FFs had significantly higher average strain values than POs did (*p* = 0.024).

**Table 6:** Strain and strain rate parameters of the study population (firefighters, police officers, and office workers).

Variables	Firefighters (FFs) (n = 97)	Police officers (POs) (n = 55)	Office workers (OWs) (n = 46)	Linear regression Estimated difference [95%-CI], *p*-value (adjusted for age)		
	Mean ± SD	Mean ± SD	Mean ± SD	OWs vs. FFs	POs vs. FFs	POs vs. OWs
Three chamber strain (%)	20.6 ± 3.7	20.1 ± 3.2	20.0 ± 2.9	−0.56 (−1.76–0.65) *p* = 0.365	−0.37 (−1.58–0.85) *p* = 0.553	0.12 (−1.11–1.35) *p* = 0.847
Four chamber strain (%)	20.4 ± 3.2	19.1 ± 2.8	20.1 ± 3.0	−0.38 (−1.46–0.70) *p* = 0.491	−1.22 (−2.23–0.21) *p* = 0.018	−1.04 (−2.22–0.14) *p* = 0.083
Two chamber strain (%)	21.1 ± 3.7	19.4 ± 3.5	21.1 ± 3.3	−0.04 (−1.28–1.19) *p* = 0.944	−1.64 (−2.81–0.47) *p* = 0.006	−1.72 (−3.10–0.35) *p* = 0.014
Average strain (%)	20.7 ± 2.8	19.6 ± 2.6	20.4 ± 2.7	−0.33 (−1.31–0.66) *p* = 0.510	−1.08 (−2.01–0.14) *p* = 0.024	−0.88 (−1.94–0.19) *p* = 0.106
LV Longitudinal Strain (%)	20.1 ± 3.1	19.5 ± 2.6	20.2 ± 2.9	0.16 (−0.86–1.19) *p* = 0.754	−0.34 (−1.22–0.54) *p* = 0.446	−0.75 (−1.84–0.34) *p* = 0.176
LV Longitudinal Strain Rate (1/s)	0.99 ± 0.21	0.95 ± 0.15	1.01 ± 0.18	0.04 (−0.03–0.11) *p* = 0.267	−0.02 (−0.08–0.03) *p* = 0.373	−0.07 (−0.13–0.00) *p* = 0.051

### Left ventricular changes

**Figure 1:**
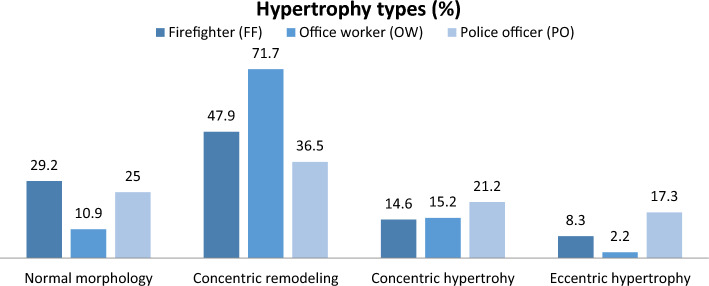
Percentage distribution of various morphological changes according to Lang et al.^24^.

Hypertrophy forms were categorized based on Lang et al.’s criteria^24^, and are illustrated in Fig. 1. Overall, significant differences were observed between FFs, POs, and OWs regarding the presence of various hypertrophy forms; the p-values of the exact Fisher test were FFs vs. OWs = 0.021, and POs vs. OWs = 0.002. A normal morphology was identified in 29.2% of FFs, 25.0% of POs, and 10.9% of OWs. OWs showed a higher incidence of concentric remodeling compared to FFs (71.77% vs. 47.9%, respectively). Concentric and eccentric hypertrophy forms were most commonly detectable in POs, with eccentric hypertrophy representing the minor standard change at 17.3%.

**Table:** 

	Firefighters (FFs) (n = 96)	Police officers (POs) (n = 52)	Office workers (OWs) (n = 46)	Criteria of Lang et al.^24^
Normal morphology	28 (29.2%)	13 (25%)	5 (10.9%)	RWD ≤ 0.42 cm and LVMM/Body surface ≤ 115 g/m^2^
Concentric remodeling	46 (47.9%)	19 (36.5%)	33 (71.7%)	RWD > 0.42 cm and LVMM/Body surface ≤ 115 g/m^2^
Concentric hypertrophy	14 (14.6%)	11 (21.2%)	7 (15.2%)	RWD > 0.42 cm and LVMM/Body surface > 115 g/m^2^
Eccentric hypertrophy	8 (8.3%)	9 (17.3%)	1 (2.2%)	RWD ≤ 0.42 cm and LVMM/Body surface > 115 g/m^2^
*p*-values (exact Fisher-Test): FFs vs. OWs, *p* = 0.021; POs vs. OWs, *p* = 0.002; FFs vs. POs, *p* = 0.223				

## Discussion

### Firefighters

The literature reports cardiac disease as the leading cause of death among American FFs, possibly due to enormous cardiovascular loads during fire-fighting missions^11^. These findings have prompted calls for regular check-up examinations and the implementation of preventive measures in the USA^28^. To date, limited published data exist regarding examinations of FFs’ cardiac structural and functional parameters. Nevertheless, echocardiography is mentioned as an essential noninvasive tool for assessing FFs’ elevated LV-hypertrophy^29^. Korre et al.^29^ demonstrated that the common occurrence of LV-hypertrophy among US firefighters plays a significant role in firefighters’ sudden cardiac death. Additionally, left ventricular mass strongly predicts cardiovascular disease events in these occupational groups. Furthermore, when the SCD autopsy results were compared with those from other firefighters who died of noncardiac causes, the SCD cases were more obese, had significantly greater heart weights, and had an increased risk of cardiomegaly (heart weight > 450 g). Two thirds of the SCD cases had a heart weight > 450 g and > 40% weighed > 550 g^30^. Our FFs cohort exhibited a high incidence of concentric structural cardiac changes. However, in the absence of long-term follow-up data, we refrain from making any assertions regarding its prognostic value for sudden cardiac death.

### Office workers

Only a few studies have investigated OWs’ cardiac function. Shirvani et al.^31^ conducted an intervention study involving a cohort of Iranian office workers. The study’s participants underwent echocardiographic assessments before and after each high-intensity interval training intervention session; a notable decrease in ESVs and increases in E/A ratios, ESDs, and EDDs following high-intensity interval training (HIIT) (in two to three weekly training sessions) was observed. These findings suggest structural adaptations in the left ventricle and enhanced LV function among non-athletes (even those leading sedentary lifestyles) following physical training, indicating the benefits of exercise. Employees with low occupational physical activity exhibit smaller left ventricular volumes and muscle mass than employees with higher physical activity^32^.

### Police officers

The quantification of cardiovascular disease-related on-duty POs deaths is challenging, but has been estimated to be between 7 and 10%^33,34^. Vavvarigou et al.^33^ identified that high-stress and physically demanding police operations, when compared to non-emergency or routine police activities, result in a significant elevation in the risk of sudden cardiac death. Incidents of sudden cardiac death were observed during conflicts (25%, n = 108), physical training (20%, n = 88), enforcement actions (12%, n = 53), rescue missions (8%, n = 34), routine duties (23%, n = 101), and other activities (11%, n = 57). Police officers face an extreme risk of cardiovascular issues^35^, sudden cardiac death^33^ and suffer from hypertension^36^ or metabolic syndrome up to 30%^19^. Yet, studies on left ventricular function in police officers are very rare, with descriptions only found among female police officers by our research group^37^. Despite extensive searches across all databases, there is no data on left ventricular function among male police officers, except for this study.

### The significance of hypertension

High blood pressure can trigger concentric hypertrophy or, initially, concentric remodeling. Elevated blood pressure imposes increased pressure on the left ventricle, initiating consecutive remodeling of the ventricular structure. This increased pressure leads to reduced end-diastolic volume due to heightened wall thickness and lack of an effective increase in left ventricular mass. The prevalence of left ventricle concentric changes observed in our study could be attributed to prevailing hypertonic changes in blood pressure. Early in the 1970s, Morganroth et al.^38^ reported that a prevailing volume load is more likely to result in an eccentric cardiac change, whereas concentric change primarily stems from pressure loading on the heart. We assumed that elevated blood pressure values were a determining factor for concentric cardiac changes observed in OWs (present in 87% of cases). Correspondingly, OWs also exhibited significantly larger septum thicknesses compared to FFs. Similarly, a concentric wall change can result from increased resting blood pressure and pressure loading^39^.

Over time, it progresses to concentric hypertrophy with diastolic dysfunction while maintaining normal systolic function. Subsequent to this phase, eccentric hypertrophy manifests when both diastolic and systolic functions are impaired^40^. It has been indicated that elevated resting blood pressure can lead to a pressure load, and consequently induce concentric myocardial changes^41^.

### Results of present study

Our evaluation of FFs, POs, and OWs revealed preserved systolic left ventricular function on average. Stroke volume and ejection fraction are indicative of the left heart’s systolic function. In comparison to OWs, FFs in our study exhibited higher LVEDV, LVESV, and SV. Conversely, POs demonstrated higher LVEDV, LVESV, SV, and EF scores than OWs did.

Additionally, POs showed higher left atrial volumes compared to OWs. The reason why POs exhibit a higher LA volume than POS can only be speculated upon perhaps it is a combination of individual factors, such as the tendency towards slightly more physical activity at the professional level. Higher levels of physical activity or arterial pressure (in the context of arterial hypertension) can lead to higher left atrial volumes^42^. Higher levels of physical activity or arterial pressure (in the context of arterial hypertension) can lead to higher left atrial volumes^42^.

LVEDV was significantly higher in FFs than in POs. Male POs in this study displayed higher LVIDd, LVIDs, and LWPWd values compared to a group of female POs^37^. LVEDV was significantly higher in FFs than in POs. Male POs in this study displayed higher LVIDd, LVIDs, and LWPWd values compared to a group of female POs^37^.

In addition to the ejection fraction, there are alternative markers to assess left ventricular function. Strain is another evaluation parameter for the heart’s contractility; strain indicates myocardial deformation and makes wall motion abnormalities visible. The literature characterizes strain assessment as a more sensitive left ventricular function evaluation method than ejection fraction^43,44^. We evaluated left ventricular function in FFs, POs, and OWs using strain analysis. On average, all three groups exhibited normal cardiac deformation values in four-chamber, two-chamber, and three-chamber views. No significant differences in myocardial deformation were detected between FFs and OWs. However, significant differences were observed in myocardial deformation between POs and OWs, specifically in the two-chamber view. Except for values observed in the two-chamber view, there were no significant myocardial strain differences between POs and OWs.

Regarding left ventricular morphology alterations, borderline septal and posterior wall thicknesses were observed in both FFs and OWs. Pathological changes in both groups were significantly higher compared to normal septal and posterior wall thickness variants. Severe pathological changes in posterior wall thickness were notably more frequent in OWs than in FFs. The changes in myocardial thickness in FFs or OWs may be attributed to higher levels of physical activity, as well as blood pressure playing a role. Blood pressure measurements in the present study were taken on a day off. It is possible that the blood pressure situation in everyday life is different from what McLaren described^45^. Nonetheless, the severity of left ventricles’ morphological changes distribution between groups did not reach statistical significance; the mean diameters of both groups’ left ventricles remained within the normal range across cardiac phases.

In comparison to studies by Yang et al.^30^, wherein 5% of deceased American FFs exhibited left ventricular hypertrophy, our study’s FFs cohort experienced more frequent hypertrophic changes. The prevalent types of hypertrophies were categorized based on relative wall thicknesses. Significantly different hypertrophy forms were observed among our three groups; concentric remodeling was the most prevalent across all groups. The second most frequent was normal morphology in FFs and concentric hypertrophy in OWs. Eccentric hypertrophy was the least common among all three occupational groups. The prevalent types of hypertrophies were categorized based on relative wall thicknesses. Significantly different hypertrophy forms were observed among our three groups; concentric remodeling was the most prevalent across all groups. The second most frequent was normal morphology in FFs and concentric hypertrophy in OWs. Eccentric hypertrophy was the least common among all three occupational groups. Concentric alterations in cardiac structure were most prevalent in examined FFs and POs. This could be explained by FFs’ and POs’ heightened physical activity compared to OWs primarily engaged in sedentary occupations. Additionally, blood pressure increases during FFs’ and POs’ operations^46^. Demanding occupational conditions place significant stress on the cardiovascular system, eliciting increased sympathetic activity. For instance, FFs’ alarm responses correlate with substantially elevated blood pressure, and an escalated heart rate poses up to 15 times higher risk of a cardiac event than experienced by the general population^11,28,47^. A potential cause of concentric cardiac changes in FFs and POs might stem from blood pressure peaks during emergency operations.^11,28,47^. A potential cause of concentric cardiac changes in FFs and POs might stem from blood pressure peaks during emergency operations.

POs and OWs professional groups exhibited, on average, an elevated BMI within the pre-obese range. Numerous studies suggest a correlation between obesity and increased left ventricular volume, left ventricular mass, and increased relative wall thickness (RWT)^48–50^. Examined POs demonstrated higher average body weight, BMI, and abdominal circumference values compared to OWs. Consequently, larger left ventricular mass and RWT values were plausible for POs.

All three occupational groups had consistent age-appropriate left ventricular diastolic function within the normal range^24^. This study’s POs’ and OWs’ mean E/A ratios were consistent with Nagueh et al.’s^51^ findings for participants aged 41–60 (mean ± SD, 1.28 ± 0.25). This study’s FFs displayed superior left ventricular diastolic function compared to OWs; on average, this study’s FFs also exhibited a higher E/A ratio, a parameter indicative of notably improved left ventricular diastolic function. Conversely, Smith et al.^52^ noted in that nearly two-thirds of a group of 207 American firefighters exhibited signs of subclinical cardiac dysfunction. Subclinical cardiac dysfunction in firefighters has been significantly associated with reduced cardiorespiratory fitness and insufficiently managed cardiometabolic risk factors. This study underscores the potential value of assessing diastolic function to evaluate and gauge increased cardiovascular event risk during firefighting operations. It should be emphasized that studies on cardiac structure among different professional groups such as firefighters, police officers, and civil servants are exceedingly rare. This study stands as the sole examination that echocardiographic assesses and compares these professional cohorts. Consequently, comparing it with other studies becomes problematic, as there is a scarcity of specific echocardiographic series examinations within these professional groups, beyond the ones cited.

## Conclusion

This study was an initial attempt to delineate and compare structural and functional cardiac parameters in various occupational groups. In terms of echocardiographic parameters, OWs exhibited a higher incidence of structural cardiac changes compared to FFs and POs. POs displayed significantly greater left ventricular mass than FFs and OWs did. Over 50% of participants in all occupational groups experienced alterations due to remodeling effects. The mean values of left ventricular ejection fraction were normal across examined occupational groups; notably, FFs exhibited significantly better diastolic function compared to POs.

This study’s findings suggest a correlation between occupational type and changes in structural and functional echocardiographic parameters. Consequently, more comprehensive epidemiological and observational studies are necessary to determine working conditions’ impacts on alterations in cardiac structural and functional parameters.

## Data Availability

The datasets analyzed during the current study are available from the corresponding author on reasonable request.
